# DNA Damage Induced by T-2 Mycotoxin in Human Skin Fibroblast Cell Line—Hs68

**DOI:** 10.3390/ijms241914458

**Published:** 2023-09-22

**Authors:** Edyta Janik-Karpinska, Michal Ceremuga, Marcin Niemcewicz, Ewelina Synowiec, Tomasz Sliwinski, Maksymilian Stela, Michal Bijak

**Affiliations:** 1 Biohazard Prevention Centre, Faculty of Biology and Environmental Protection, University of Lodz, Pomorska 141/143, 90-236 Lodz, Poland; edyta.janik@edu.uni.lodz.pl (E.J.-K.); marcin.niemcewicz@biol.uni.lodz.pl (M.N.); maksymilian.stela@biol.uni.lodz.pl (M.S.); 2 Military Institute of Armament Technology, Prymasa Stefana Wyszyńskiego 7, 05-220 Zielonka, Poland; michal.ceremuga@witpis.eu; 3 Laboratory of Medical Genetics, Faculty of Biology and Environmental Protection, University of Lodz, Pomorska 141/143, 90-236 Lodz, Poland; ewelina.synowiec@biol.uni.lodz.pl (E.S.); tomasz.sliwinski@biol.uni.lodz.pl (T.S.)

**Keywords:** T-2 toxin, skin, Hs68 cell line, genotoxicity, DNA

## Abstract

T-2 mycotoxin is the most potent representative of the trichothecene group A and is produced by various *Fusarium* species, including *F. sporotrichioides*, *F. poae*, and *F. acuminatum*. T-2 toxin has been reported to have toxic effects on various tissues and organs, and humans and animals alike suffer a variety of pathological conditions after consumption of mycotoxin-contaminated food. The T-2 toxin’s unique feature is dermal toxicity, characterized by skin inflammation. In this in vitro study, we investigated the molecular mechanism of T-2 toxin-induced genotoxicity in the human skin fibroblast—Hs68 cell line. For the purpose of investigation, the cells were treated with T-2 toxin in 0.1, 1, and 10 μM concentrations and incubated for 24 h and 48 h. Nuclear DNA (nDNA) is found within the nucleus of eukaryotic cells and has a double-helix structure. nDNA encodes the primary structure of proteins, consisting of the basic amino acid sequence. The alkaline comet assay results showed that T-2 toxin induces DNA alkali-labile sites. The DNA strand breaks in cells, and the DNA damage level is correlated with the increasing concentration and time of exposure to T-2 toxin. The evaluation of nDNA damage revealed that exposure to toxin resulted in an increasing lesion frequency in Hs68 cells with *HPRT1* and *TP53* genes. Further analyses were focused on mRNA expression changes in two groups of genes involved in the inflammatory and repair processes. The level of mRNA increased for all examined inflammatory genes (*TNF*, *INFG*, *IL1A*, and *IL1B*). In the second group of genes related to the repair process, changes in expression induced by toxin in genes—*LIG3* and *APEX* were observed. The level of mRNA for *LIG3* decreased, while that for *APEX* increased. In the case of *LIG1*, *FEN*, and *XRCC1*, no changes in mRNA level between the control and T-2 toxin probes were observed. In conclusion, the results of this study indicate that T-2 toxin shows genotoxic effects on Hs68 cells, and the molecular mechanism of this toxic effect is related to nDNA damage.

## 1. Introduction

Trichothecene mycotoxins are produced mainly by *Fusarium* species and are common contaminants found in various cereals, including wheat, maize, barley, and other agricultural products. Therefore, there is a major health and food safety concern due to their toxicity to both humans and animals [1]. T-2-toxin is the most toxic representative of the trichothecene group A and is produced by different *Fusarium* species such as *F*. *sporotrichioides*, *F*. *acuminatum*, and *F*. *poae*. It is extremely chemically stable under various environmental conditions, such as high temperatures and UV light. What is more, T-2 toxin inactivation during feed production and processing is not always effective. The T-2 toxin’s lipophilic nature suggests that it is easily absorbed through the intestines, pulmonary mucosa, and skin [2]. Numerous in vitro and in vivo studies showed that exposure to T-2 toxin can cause various organ damage, including liver [3], kidney [4], intestines [5], brain [6], and reproductive system. In addition, unlike most biological toxins, T-2-mycotoxin is characterized by possessing a highly irritating effect on the skin [7]. In our previous study [8] performed on the human skin fibroblast Hs68 cell line, the T-2 toxin cytotoxic effect was demonstrated. This study clearly indicated that the T-2 toxin induces necrosis as a toxicity effect in an in vitro human skin model. A variety of mechanisms of action have been proposed for T-2 toxin. At the molecular level, T-2 toxin inhibits protein synthesis by binding peptidyl transferase, which is an integral part of the 60S ribosomal subunit. The inhibition of DNA and RNA biosynthesis by T-2 toxin was also observed [9]. T-2 toxin interferes with the metabolism of the phospholipid membrane and increases the level of liver lipid peroxides [10]. Studies showed that toxin also inhibits mitochondrial function, the mitochondrial electron transport system, and mitochondrial protein synthesis [11]. In vitro studies also revealed cell membrane disruption and the toxic effects of cell division and proliferation after T-2 toxin exposure [12].

A number of studies have suggested that T-2 mycotoxin may induce DNA damage and apoptosis. Chaudhari et al. investigated the molecular mechanisms of DNA damage induced by T-2 toxin and apoptosis in the human cervical cancer (HeLa) cell line. The obtained results showed increased levels of mitochondrial apoptogenic factors Bax, Bcl-2, and cytochrome-c followed by activation of caspases-3, -7, and -9, leading to DNA fragmentation and apoptosis. Moreover, the caspase-independent apoptosis-inducing factor (AIF) pathway also leads to DNA fragmentation and apoptosis in T-2 toxin-treated HeLa cells [13]. In different studies, the impact of T-2 toxin on reproductive cells using TM3 Leydig cells was investigated. DNA condensation and fragmentation were clearly observed in TM3 Leydig cells treated with T-2 toxin. According to the results, T-2 toxin inhibited cell proliferation and induced cell membrane and DNA damage, which led to increased apoptosis in treated cells [14]. An in vivo study provided evidence that T-2 toxin is genotoxic to pig lymphocytes at a concentration of 3 mg/kg feed, leading to lymphocyte DNA fragmentation [15]. Similar results were obtained in an experiment on chickens, where T-2 toxin caused DNA fragmentation in spleen leukocytes when added at a concentration of 10 mg/kg feed [16]. Different study results indicated dose-dependent impaired performance and DNA fragmentation in spleen leukocytes of broiler chickens induced by T-2 toxin [17]. Szabo et al. analyzed the T-2 toxin genotoxic effect in broiler chicken hepatocytes. The aim of this study was to evaluate the effect of mycotoxin on DNA damage in chicken liver cells. Results showed the potential T-2 toxin DNA-damaging effect after 14 days of exposure [18].

Although T-2 toxin has adverse effects on different tissues and organs, its molecular effects and mechanism of action on the skin still remain unknown. The objective of this study was to investigate whether T-2 effects resulted in DNA damage in an in vitro model using the normal human fibroblast Hs68 cell line.

## 2. Results

### 2.1. DNA Damage—Comet Assay

**Figure 1:**
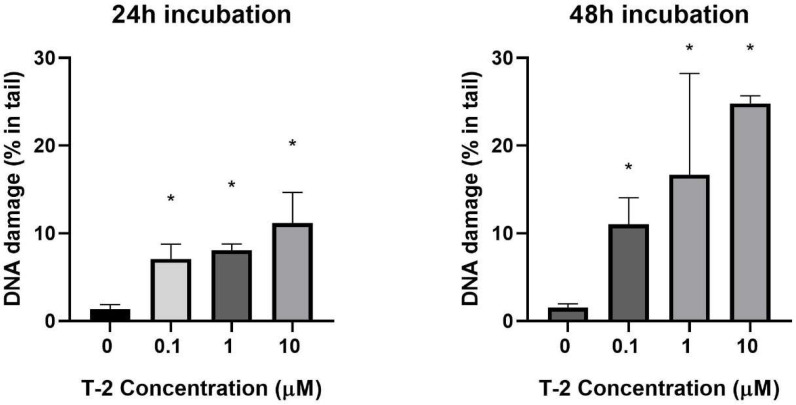
The mean level of DNA strand breaks, alkali-labile sites, and oxidative DNA damage induced by T-2 toxin in Hs68 cells. Values present are means ± SD (*n* = 6). * *p* < 0.05.

The level of DNA damage induced by T-2 toxin in the Hs68 cell line was determined using an alkaline version of the comet assay, which measures the amount of DNA at alkali-labile sites and strand breaks. Significantly higher DNA damage was noticed after treatment of the Hs68 cells with T-2 toxin (*p* < 0.05) (Figure 1). The increasing DNA damage has been demonstrated by an increasing percentage of DNA in the comet tail and a decreasing percentage of DNA in the head. The dose-dependent manner as well as the time-dependent manner were observed.

### 2.2. Determination of Nuclear DNA Damage—Semi-Long Run qRT-PCR (SLR-qRT-PCR)

**Figure 2:**
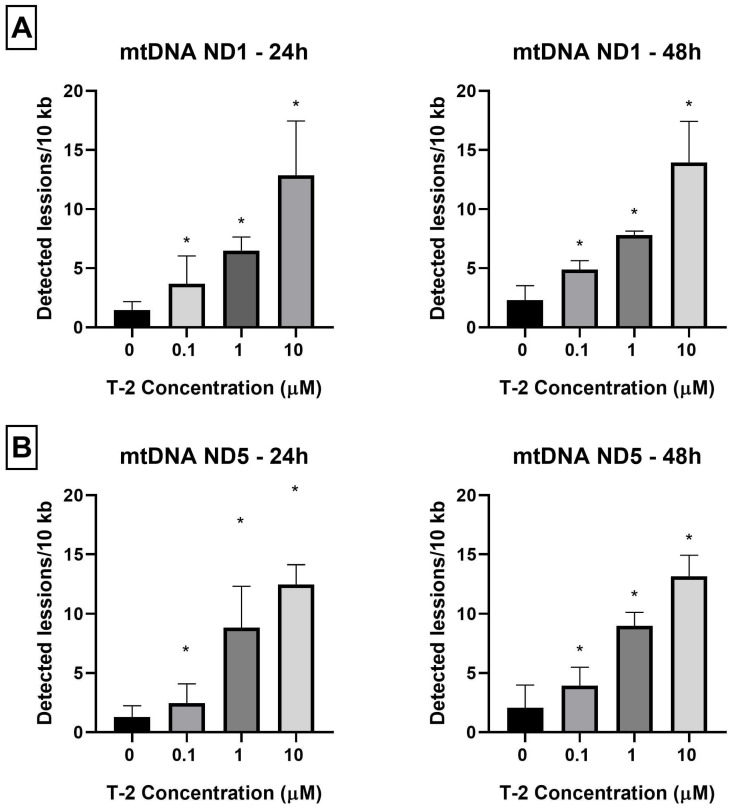
(**A**,**B**) The effect of T-2 toxin on nDNA lesion frequency per 10 kb of DNA in *HPRT1* and *TP53* genes was estimated by SLR-qRT-PCR amplification of total DNA from Hs68 cells. Values present are means ± SD (*n* = 6). * *p* < 0.05.

As a next evaluation of T-2 toxin genotoxicity, the determination of nDNA damage in the *HPRT1* and *TP53* regions was performed. The nDNA damage caused by SLR-qRT-PCR amplification of DNA isolated from cells exposed to T-2 toxin at concentrations of 0.1, 1, and 10 μM in two incubation periods—24 h and 48 h—was examined. It was observed that treatment of Hs68 cells by T-2 toxin in a dose- and time-dependent manner increased the level of nDNA damage in both tested nDNA regions—hypoxanthine-guanine phosphoribosyltransferase 1 (*HPRT1*) (Figure 2A) and the tumor suppressor protein p53 (*TP53*) (Figure 2B). No significant difference in the level of damage was found between the evaluated regions. In both tested genes, in the case of probes treated with T-2 toxin at a concentration of 10 μM after 48 h, the maximum of calculation possibilities (the highest attempts at measurements) were obtained.

### 2.3. Analysis of Gene Expression

**Figure 3:**
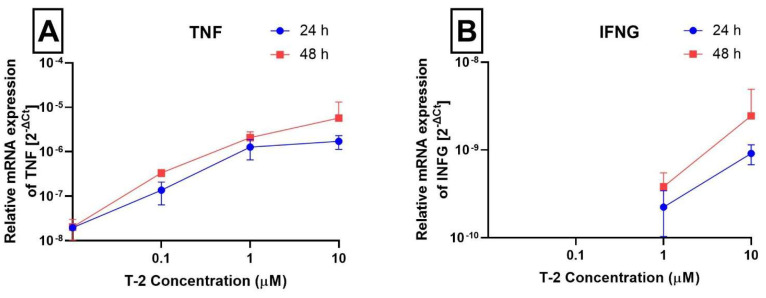

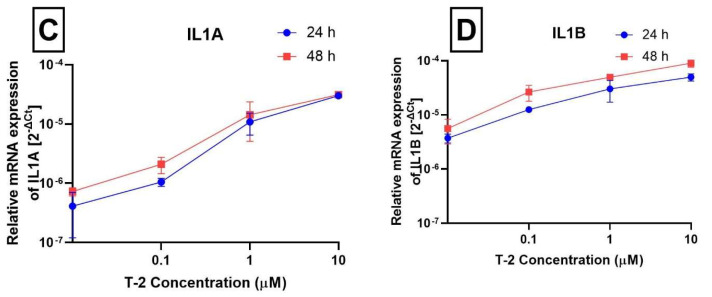
The effect of T-2 toxin on the expression of *TNF* (**A**), *IFNG* (**B**), *IL1A* (**C**), and *IL1B* (**D**) genes (measured at the mRNA level). The results are expressed as a mean of 2^−Ct^ (according to the reference gene—*18S rRNA*) ± SD, *n* = 6.

In order to evaluate the effect of T-2 toxin on the expression of important DNA damage genes, a gene expression analysis at the mRNA level using the Real-Time PCR method was performed. The evaluation of changes in the mRNA expression of two groups of genes was executed. First were the inflammatory genes: Tumor Necrosis Factor (*TNF*), Interferon Gamma (*INFG*), Interleukin 1 alpha (*IL1A*), and Interleukin 1 beta (*IL1B*). The second group was related to the DNA repair process—DNA LIGASE 1 (*LIG1*), DNA LIGASE 3 (*LIG3*), Flap Structure-Specific Endonuclease (*FEN*), X-Ray Repair Cross Complementing 1 (*XRCC1*), and Apurinic/Apyrimidinic Endodeoxyribonuclease (*APEX*). The results presented in Figure 3A–D demonstrate that T-2 toxin in a dose- and time-dependent manner increased the level of mRNA for *TNF, INFG, IL1A*, and *IL1B*. In the case of the *INFG* gene, it was detected in probes without T-2 toxin and probes treated with T-2 toxin at the lowest concentration (0.1 μM); however, the mRNA was detected in probes treated with T-2 toxin at the lowest concentrations of 1 and 10 μM in both incubation periods—24 h and 48 h (Figure 3B).

**Figure 4:**
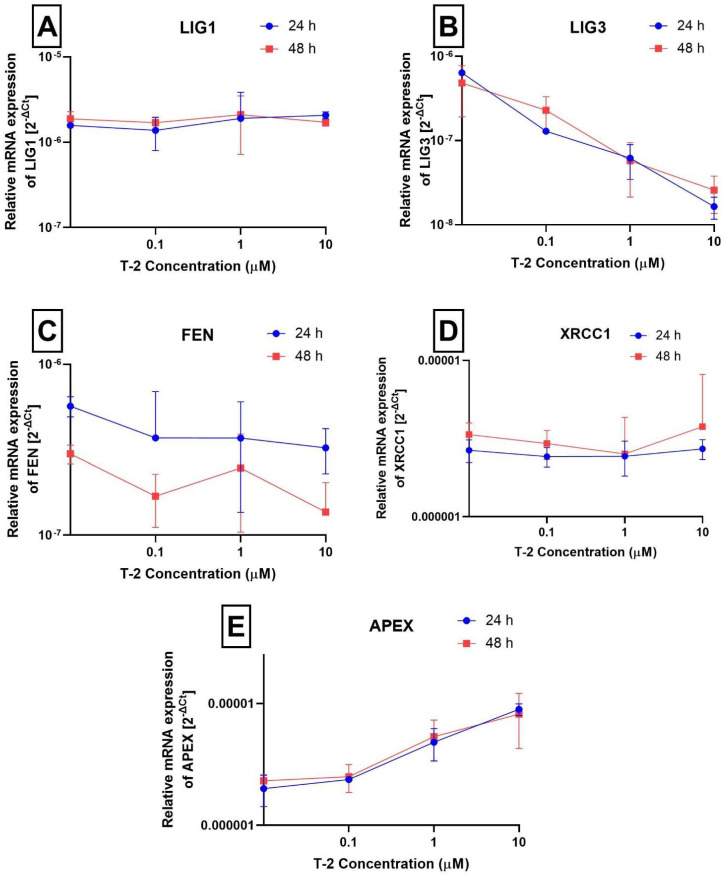
The effect of T-2 toxin on the expression of *LIG1* (**A**), *LIG3* (**B**), *FEN* (**C**), *XRCC1* (**D**), and *APEX* (**E**) genes (measured at the mRNA level) The results are expressed as a mean of 2^−ΔCt^ (according to the reference gene—*18S rRNA*) ± SD, *n* = 6.

In the next group of genes (Figure 4A–E), the changes in expression induced by T-2 toxin in two of them—*LIG3* and *APEX*—were observed. The level of mRNA for *LIG3* is decreasing in a dose-dependent manner (Figure 4B), while for APEX, the level is increasing (Figure 4E). In the cases of *LIG1* (Figure 4A), *FEN* (Figure 4C), and *XRCC1* (Figure 4D), there were no changes in mRNA levels between the control and T-2 toxin probes.

## 3. Discussion

The T-2 toxin possesses harmful toxic effects on humans and animals [12,19]. Although various studies have examined DNA damage in different in vitro and in vivo models [3,13,20,21], no reports have focused on the effects of T-2 toxin on human skin. Herein, we report that T-2 toxin induces nuclear DNA damage in human Hs68 cell lines.

DNA is the basic unit of inheritance and a template for the processes of replication and transcription, making the maintenance of genetic stability crucial for viability. It is also an intrinsically reactive molecule and is highly susceptible to chemical modifications by various endogenous and exogenous (environmental) agents [22]. DNA damage is a common phenomenon for every cell during its lifetime and is defined as a change in the chemical structure of genomic DNA. Most of the endogenous DNA damage arises from the chemically active DNA involved in oxidative and hydrolytic reactions with reactive oxygen species (ROS) and water, respectively, that are naturally present within cells [23]. Cells may also accumulate somatic genome changes due to errors during DNA replication in the form of small base insertions or deletions (indels), substitutions, and gross chromosomal rearrangements. DNA-damaging events caused by endogenous agents generally occur much more frequently than damage caused by exogenous agents [24]. The sources of exogenous DNA damage are ionizing radiation (IR), ultraviolet (UV), and various chemical agents [25]. IR is a type of high-energy radiation that is able to release electrons from atoms and molecules, generating ions that can break covalent bonds. IR directly affects DNA structure by inducing DNA breaks, in particular DNA double-strand breaks (DSBs). The secondary effect is ROS generation, which oxidizes lipids and proteins and also induces several DNA damages, such as the generation of basic sites and single-strand breaks (SSBs). Taking them together, these changes induce mitotic failure and cell death [26]. UV is a form of non-ionizing radiation that is emitted by the sun and different artificial sources such as tanning beds, some halogen, fluorescent, and incandescent lights, or mercury vapor lighting [27]. UV radiation causes DNA damage indirectly by producing ROS and directly by the covalent modification of neighboring pyrimidines. Two major UV-induced DNA lesions are cyclobutane pyrimidine dimers and a 6-4 photoproduct that is characterized by the formation of a covalent bond between two adjacent pyrimidine bases: C6 of the 5’-base and C4 of the 3’-base. DNA damage induced by UV radiation causes genetic alterations, resulting in mutagenesis and cancer formation [28]. Another type of exogenous DNA damage is caused by alkylating agents. Major sources of external alkylating agents include industrial processing, tobacco smoke, chemotherapeutic agents, and dietary components. The cytotoxic effect of alkylating agents is mainly due to the alkylation of DNA bases, which can interfere with essential DNA processes such as DNA replication and/or transcription [23]. Alkylating agents react with the nitrogen’s (N) ring and extracyclic oxygen (O) atoms of DNA bases to form a variety of covalent adducts ranging from simple methyl groups to complex alkyl additions. DNA damage generated by these agents depends on their specific chemical reactivity (S_N_1-type or S_N_2-type nucleophilic substitution), the number of reactive sites in the alkylating agent (monofunctional or bifunctional), the type of alkyl group addition, and the DNA substrate (single-stranded or double-stranded) [29]. Monofunctional agents contain one active chemical moiety to modify a single site in DNA, while bifunctional agents contain two reactive groups that can bond to separate DNA bases, forming interstrand crosslinks. S_N_2-alkylating agents mainly target ring nitrogen atoms in DNA bases, whereas S_N_1-alkylating agents can modify these nitrogen and the extracyclic oxygen groups. Depending on the nature of the nucleophile and the alkylating agent, preferential sites of alkylation in DNA can be distinguished. Generally, base alkylation mainly occurs at positions guanine N7 and O6, adenine N1 and N3, and cytosine N3. Most of the monofunctional methylating agents induce the formation of N7-methylguanine (7meG) as the dominant methylation adduct, which accounts for 60–80% of all alkylation changes in DNA. 7meG does not have any cytotoxic or mutagenic properties; however, it is prone to spontaneous depurination, forming an apurinic (AP) site that is toxic and mutagenic. Monofunctional methylating agents can also generate N3-methyladenine (3meA) as the other N-methylation product, which accounts for 10–20% of all methyl adducts. The 3meA is highly cytotoxic, as it can block most DNA polymerases and thus inhibit the synthesis of DNA [23,30]. Among the DNA oxygen atoms, the O6 position of guanine is the primary site of methylation by S_N_1-type agents for generating O6-methylguanine (O6meG). Induction of O6meG lesions is of crucial biological importance, as O6meG can easily mispair with thymine during DNA replication, causing many of the cytotoxic and mutagenic effects [30]. Sulfur and nitrogen mustards are highly reactive, bifunctional alkylating agents. These compounds react readily with N7-guanine and, to a lesser extent, with N3-adenine and N7-adenine, forming N-monoadducts. Then, these monoadducts can react with another base to form guanine–guanine (G–G) and guanine–adenine (G–A) inter-strand crosslinks [31].

This study is a continuation of our in vitro research focused on the T-2 mechanism of action in a human skin model. In our previous study [8] we demonstrated the necrosis potential of T-2 toxin with a strong reduction of ATP production by cells. We also determined the impact of T-2 toxin on mitochondrial physiology and disruption of mitochondrial DNA (mtDNA) [32]. This study focuses on the nDNA damage of the human normal fibroblast cell line—Hs68 as a result of exposure to T-2 toxin.

The comet assay (single-cell gel electrophoresis) is a method for measuring DNA damage in eukaryotic cells. There are two types of comet assays. A neutral comet assay is used to detect DNA double-strand breaks (DSBs). The alkaline version of the comet assay is the most widely used and is appropriate for smaller amounts of DNA damage, including DNA single-strand breaks (SSBs) and DSBs, SSBs associated with incomplete excision repair sites, alkali-labile sites, and DNA-DNA or DNA-protein cross-linking. Both versions allow visualization of fragmented DNA and provide a simple way to quantify DNA damage [33] A comet assay is considered a sensitive method for in vitro and in vivo genetic toxicological studies and is also suitable for various research areas, such as molecular biology [34], environmental monitoring [35] and medicine [36]. In various studies, the genotoxic effect of T-2 toxin was measured by a comet assay. In the Szabo et al. [18] study, a comet assay was used to investigate the DNA-damaging effects of T-2 and HT-2 mycotoxins in the liver of broiler chickens. Results showed that the comet assay was successfully adapted to chicken hepatocytes, and the DNA damage was determined by a decrease in the DNA percentage in the comet tail in the experimental groups. Each toxin-treated group differed significantly from the control group, indicating that this assay can be useful for the evaluation of primary DNA damage caused by T-2/HT-2 mycotoxins. In different studies, the rate of DNA damage caused by T-2 toxin in healthy porcine mononuclear cells was assessed. As T-2 toxin concentration increased, the frequency of intact lymphocytes decreased from 50.2% (0.1 μM) to 36.3% (1.0 μM) within the first 24 h. It was concluded that a dose- and time-dependent DNA-damaging effect of T-2 toxin could be demonstrated using peripheral blood mononuclear cells from porcine by an alkaline comet assay [37]. However, this is the first study in which the normal human fibroblast cell line—Hs68 exposed to T-2 toxin was used in a comet assay to detect the time- and concentration-dependent DNA-damaging effects. Using this technique, we evaluated the rate of DNA damage caused by T-2 toxin in increasing concentrations after 24 h and 48 h of incubation. Our results showed that T-2 toxin induces DNA alkali-labile sites and strand breaks in the Hs68 cell line. The level of DNA damage in cells is correlated with the level and time of T-2 toxin exposure. These results confirm that T-2 toxin is toxic to fibroblast cells, and this toxicity includes DNA damage.

The *HPRT1* (hypoxanthine-guanine phosphoribosyltransferase) gene is believed to be a housekeeping gene and is the most frequently analyzed in DNA damage studies. The protein encoded by this gene is an enzyme transferase, which plays a crucial role in the generation of purine nucleotides through the purine salvage pathway. The HPRT1 enzyme recycles nucleotides to supply DNA and RNA synthesis in vital and actively dividing cells, explaining the great presence of HPRT1 in most tissues. It is considered that HPRT1 functions in multiple housekeeping roles, including cell cycle and proliferation mechanisms, DNA replication and repair, and RNA metabolism [38]. The *TP53* gene encodes a TP53 protein containing transcriptional activation, DNA binding, and oligomerization domains. The TP53 protein provides substantial functions in the cellular response to various stresses and ensures the maintenance of genome integrity. It responds to diverse cellular stresses to regulate the expression of target genes, hence inducing DNA repair, cell cycle arrest, apoptosis, or metabolic adaptations [39]. Both of these genes play an essential role in cell function. In this study, we evaluated the effect of T-2 toxin on nDNA lesion frequency in *HPRT1* and *TP53* genes in Hs68 cells. According to the results, T-2 toxin increases DNA lesions in the evaluated regions in a dose- and time-dependent manner. It confirms that T-2 toxin has a toxic effect on fibroblast skin cells, and this effect includes nDNA lesions.

A number of cytokines, especially Interleukin 1 (IL1) and tumor necrosis factor (TNF), play a significant role in initiating the inflammatory process. They are secreted by different cells, including macrophages, monocytes, and adipocytes. Together with various cytokines and growth factors, such as Interleukin 8 (IL8) and the granulocyte-macrophage colony-stimulating factor (GM-CSF), they induce gene expression and protein synthesis thus mediating the onset and development of the inflammation process. IL1A and IL1B, which belong to the group of cytokines, which are elements of innate and adaptive immunity, act as mediators of inflammation. Interleukins are classified as proteins and are mainly synthesized by leukocytes, although they can also be produced by non-immune cells such as endothelial cells in response to inflammation. Despite the significant similarities of both interleukins in structure, biological activity, and the same type of receptor, the key difference between both ILs is the place where they interact with other cells. IL1A associates with the cell membrane of the cells in which it is produced, from where it affects the cells in the immediate vicinity, while IL1B, synthesized in the form of pro-interleukin, which requires caspase 1 to activate, is secreted into the blood, acting systemically [40]. The level of gene expression of both ILs, and thus the activity of cytokines, is low in the case of good health and increases under the influence of inflammation. The increase in gene expression of both ILs suggests that T-2 toxin induces inflammation in Hs68 cells. A particularly significant increase in expression is observed in the *IL1B* gene, which may indicate that the inflammation induction associated with cell DNA damage, resulting in the cell’s necrotic pathway, may affect other, distant cells in the context of the entire organism.

The *APEX* gene is responsible for encoding the major human endonuclease AP sites, resulting in DNA damage. In the case of DNA replication containing AP sites, random insertion of a nucleotide may occur during the synthesis of a new strand of DNA. To eliminate replication errors that could result in mutation, cells have developed a system to identify and repair AP sites. AP endonuclease leads to cleavage of the phosphodiester backbone from the 5’ end towards the AP site, initiating base excision repair (BER) [23]. The APEX protein is ubiquitous in the organism; however, variable levels have been found in various types of cancer, such as hepatocellular carcinomas, breast carcinomas, rhabdomyosarcomas, or gastric carcinomas. According to the Kim et al. [41] study, results on the expression of *APEX* as a potential diagnostic biomarker of clear cell renal cell carcinoma and hepatobiliary carcinomas suggest active extracellular secretion of APEX from cancer cells, activated stromal cells, and inflammatory cells. Thus, it can be seen that *APEX* gene expression increases with the level of DNA damage. *LIG3* encodes a protein functioning in DNA replication and repair pathways, including nucleotide excision repair, base excision repair, and single-strand break repair. *LIG3* is also involved in the repair of DNA double-strand breaks when nonhomologous end joining (NHEJ) activity is impaired. The *LIG3* gene is ubiquitously expressed at low levels in almost all human tissues and cells [42]. Treatment of Hs68 cells with T-2 toxin at appropriate concentrations resulted in an increase in *APEX* gene expression, which indicates an increase in the cell’s DNA damage caused by the appearance of AP sites. A decrease in *LIG3* gene expression can be a result of substantial cell damage and, as a consequence, impaired DNA repair mechanisms.

## 4. Materials and Methods

### 4.1. Reagents

Dimethyl sulfoxide (DMSO), T-2 Toxin from *Fusarium* sp. (cat. No. T4887), DAPI (4′,6-diamidino-2-phenylindole), and low- and normal-melting-point agarose were obtained from Sigma-Aldrich Chemical Co. (St. Louis, MO, USA). Penicillin-Streptomycin mixture, Dulbecco’s Modified Eagle Medium (DMEM) with 4.5 g/L Glucose and L-Glutamine, and fetal bovine serum (FBS) heat-inactivated PBS (1X) without calcium or magnesium were purchased in Lonza (Basel, Switzerland). The High-Capacity cDNA Reverse Transcription Kit, TaqMan probes, and Universal Master Mix were obtained from Life Technologies (Grand Island, NY, USA). All other chemicals were reagent-grade or the highest-quality available.

### 4.2. Cell Culture and Treatment

The human foreskin fibroblast line Hs68 (ATCC^®^ CRL-1635™) was obtained from the American Type Culture Collection (ATCC™, Manassas, VA, USA). Hs68 cells were cultured in DMEM supplemented with 100 units of potassium penicillin, 100 μg of streptomycin sulfate per 1 mL of culture media, and 10% (*v*/*v*) FBS. Cultures were maintained at 37 °C in a humidified atmosphere with 5% CO_2_. To perform analysis, cells were seeded at 3 × 10^6^ cells and left in an incubator for 12 h before treatment procedures. Next, the cell samples were incubated with T-2 toxin in a concentration range of 0.1 to 10 μM for 24 h and 48 h. The working solutions for T-2 toxin were made by direct dilution of the toxin in culture medium. The untreated cells were used as a control.

### 4.3. DNA Damage—Comet Assay

DNA damage in the Hs68 cell line was estimated by the alkaline version (pH > 13) of the comet assay [43]. A freshly prepared suspension of cells in 0.75% low melting point agarose dissolved in phosphate buffered saline was cast on microscope slides precoated with 0.5% normal melting agarose, covered with a cover glass, and placed on a cold plate for 10 min. Next, cover glass was removed, and cells were lysed for 1 h at 4 °C in a buffer consisting of 2.5 M NaCl, 100 mM EDTA, 1% Triton X-100, 10 mM Tris, pH 10. Then, DNA was allowed to unwind for 20 min in an electrophoretic solution consisting of 300 mM NaOH and 1 mM EDTA. Electrophoresis was conducted for 20 min at an electric field strength of 0.73 V/cm (30 mA) in a buffer of pH > 13 (300 mM NaOH and 1 mM EDTA). After electrophoresis, slides were washed twice with deionized water and left to dry out. For analysis, samples were stained for at least 60 min with 2 μg/mL DAPI and covered with cover slips. Then, the comet pictures were captured at 200× magnification in an Eclipse fluorescence microscope (Nikon, Tokyo, Japan) with a COHU 4910 video camera (Cohu, Inc., San Diego, CA, USA) equipped with a UV filter block consisting of an excitation filter (359 nm) and barrier filter (461 nm) and connected to an image analysis system, Lucia-Comet v. 4.51 (Laboratory Imaging, Prague, Czech Republic). For each sample, 50 comets were randomly selected, and the comet’s tail DNA was measured. Two parallel tests with aliquots of the same sample of cells were performed for a total of 100 cells. Each experiment was repeated twice. The percentage of DNA in the tail (% tail DNA) was analyzed. The mean value of the tail DNA in the sample was taken as an index of DNA damage.

### 4.4. Isolation of Total Genomic DNA from Cell Lines

Total genomic DNA (mitochondrial and nuclear) from the cell pellets was isolated by using the commercially available EXTRACT ME RNA and DNA KIT (BLIRT S.A., Gdansk, Poland), according to the producer’s protocol. DNA concentrations were determined by spectrophotometric measurement of the absorbance at 260 nm. The purities were calculated by an A260/A280 ratio using the Bio-Tek Synergy HT Microplate Reader (Bio-Tek Instruments, Winooski, VT, USA). The purified DNA was stored at −30 °C until further analysis.

### 4.5. Determination of Nuclear DNA Damage—Semi-Long Run qRT-PCR (SLR-qRT-PCR)

The assessment of nuclear DNA (nDNA) damage was performed using semi-long-run quantitative RT-PCR (SLR-qRT-PCR). The levels of DNA lesions in the tested region of the nuclear genome were measured using two fragments of different lengths, i.e., small and long fragments, located in the same nuclear genomic region. The sequence of all primers used in this study is listed in Table 1. All primers were designed using the Primer3 software (http://bioinfo.ut.ee/primer3-0.4.0/, accessed on 5 August 2022) and synthesized by Sigma-Aldrich (St. Louis, MO, USA). Complete nucleotide sequences for each gene were taken from the ENSEMBL database (https://ensembl.org/, accessed on 5 August 2022).

SLR-qRT-PCR amplification was performed using a CFX96 Real-Time PCR Detection System (Bio-Rad Laboratories, Hercules, CA, USA). The SLR-qRT-PCR reaction mix in a total volume of 10 μL included 1 × Power SYBR Green PCR Master Mix (Thermo Fisher Scientific, Waltham, MA, USA), 250 nM of each primer, and 5 ng of template DNA. The PCR reaction conditions were as follows: enzyme activation at 95 °C for 10 min, followed by up to 40 cycles of 15 s denaturation at 95 °C, 30 s annealing at 65 °C, and 15 s extension at 72 °C (for short amplicons) or 45 s at 72 °C (for long amplicons). The Ct values were computed automatically and subsequently analyzed using the CFX Manager^TM^ Software (version 3.1). DNA damage was calculated as a lesion per 10 kb of DNA in each region by including the size of the particular long fragment. The following formula was used: lesion per 10 kb DNA = (1 − 2 − (Δlong − Δshort)) × 10,000 (bp)/size of long fragment (bp), where ∆long and ∆short show differences in Ct value between treated samples and non-treated cells (control). DNA isolated from controls was used as a reference, while the CT of large and small nuclear fragments was used for DNA damage quantification.

### 4.6. Gene Expression Analysis Using Real-Time PCR

Total RNA from the cell pellets was extracted using EXTRACT ME RNA and DNA KIT (BLIRT S.A., Gdansk, Poland), according to the manufacturer’s protocol. RNA purity and concentration were determined by comparing the absorbances at 260 and 280 nm using the Bio-Tek Synergy HT Microplate Reader (Bio-Tek Instruments, Winooski, VT, USA). RNA was stored at −30 °C until further analysis. cDNA was synthesized from total RNA using the High-Capacity cDNA Reverse Transcription Kit. A sample of 2 μg of total RNA was used as a template in a total volume of 10 μL. Gene expression was analyzed by a TaqMan probe-based real-time PCR assay. TaqMan^®^ gene expression details are included in Table 2.

Reactions were carried out in a thermal cycler CFX96™ Real-Time PCR Detection System (Bio-Rad Laboratories, Hercules, CA, USA). The thermal cycling conditions were as follows: a total of 10 min of polymerase activation at 95 °C, followed by 40 cycles of 30 s denaturation at 95 °C and 60 s annealing/extension at 60 °C. Each sample was run in triplicate. The cycle threshold (Ct) values were calculated automatically by CFX96™ Real-Time PCR Detection System equipped with CFX Manager TM Software (version 3.1). Relative expressions of the studied genes were calculated using the equation 2^−ΔCt^, where ΔCt = Ct target gene − Ct18S rRNA.

### 4.7. Data Analysis

All obtained experimental values were elaborated using Microsoft Excel software (Redmond, WA, USA, https://www.microsoft.com/en-us/microsoft-365/excel, accessed on 5 August 2022) and stated as mean values ± standard deviations (SD). The statistical analysis procedures were made using StatsDirect statistical software V. 2.7.2. (Cheshire, UK). All data were analyzed using the Shapiro-Wilk test for normality. The results were examined according to equality of variance via Levene’s test. The significance of the differences among the values was analyzed using ANOVA: Tukey’s range test (for data with a normal distribution and significant equality of variance) or the Kruskal-Wallis test; *p* < 0.05 was accepted as statistically significant [44,45].

## 5. Conclusions

We demonstrated for the first time that in an in vitro human skin fibroblast model, T-2 toxin shows genotoxic properties. Results presented in this study showed that T-2 toxin exposure causes DNA fragmentation, decreases the expression of repair process genes, and increases the expression of inflammatory genes. The molecular mechanism of this toxic effect is related to nDNA damage, leading to impaired cellular function and cell loss.

## Figures and Tables

**Table 1:** SLR-qRT-PCR primers used for quantification of nuclear DNA damage.

Target Gene Symbol	Forward Primer Sequences (5′→3′)	Reverse Primer Sequence (5′→3′)	Amplicon Length (bp)
*TP53* (tumor protein p53)	Long fragment: GGGTGTAGATGATGGGGATG	Long fragment: AACTGCGGAATGAAACAACC	1172
	Small fragment: AAGCTGCTAAGGTCCCACAA	Small fragment: GGAAAGATCGCTCCAGGAA	56
*HPRT1* (hypoxanthine phosphoribosyltransferase 1)	Long fragment: AGGGCAAAGGATGTGTTACG	Long fragment: AGTGGTTTCTGGTGCGACTT	1018
	Small fragment: TGCTGACCTGCTGGATTACA	Small fragment: TCTACAGTCATAGGAATGGATCTATCA	69

**Table 2:** TaqMan^®^ Gene Expression Assays details used in this study.

Gene Name	Gene Symbol	Entrez Gene ID	RefSeq	TaqMan^®^ Gene Expression Assay IDs *
Eukaryotic 18S rRNA	*18S rRNA*	*HSRRN18S*	X03205.1 (GenBank mRNA)	Hs99999901_s1
Tumor necrosis factor	*TNF*	7124	NM_000594.3	Hs00174128_m1
Interferon gamma	*INFG*	3458	NM_000619.2	Hs00989291_m1
Interleukin 1 alpha	*IL1A*	3552	NM_000575.4	Hs00174092_m1
Interleukin 1 beta	*IL1B*	3553	NM_000576.2	Hs01555410_m1
DNA ligase 1	*LIG1*	3978	NM_000234.2	Hs01553527_m1
DNA ligase 3	*LIG3*	3980	NM_002311.4	Hs00242692_m1
Flap structure-specific endonuclease 1	*FEN1*	2237	NM_004111.5	Hs00748727_s1
X-ray repair cross complementing 1	*XRCC1*	7515	NM_006297.2	Hs00959834_m1
Apurinic/apyrimidinic endodeoxyribonuclease 1	*APEX1*	328	NM_001244249.1	Hs00959050_g1

* Obtained from Applied Biosystems (Thermo Fisher Scientific).

## Data Availability

Not applicable.
